# ID-Seg: an infant deep learning-based segmentation framework to improve limbic structure estimates

**DOI:** 10.1186/s40708-022-00161-9

**Published:** 2022-05-28

**Authors:** Yun Wang, Fateme Sadat Haghpanah, Xuzhe Zhang, Katie Santamaria, Gabriela Koch da Costa Aguiar Alves, Elizabeth Bruno, Natalie Aw, Alexis Maddocks, Cristiane S. Duarte, Catherine Monk, Andrew Laine, Jonathan Posner, program collaborators for Environmental influences on Child Health Outcomes

**Affiliations:** 1 Department of Psychiatry and Behavioral Sciences, Duke University, Durham, NC USA; 2 New York State Psychiatric Institute, New York, NY USA; 3 Department of Biomedical Engineering, Columbia University, New York, NY USA; 4 Department of Computer Science, University Of Toronto, Toronto, ON Canada; 5 Department of Obstetrics and Gynecology, Columbia University, New York, NY USA; 6 Department of Radiology, Columbia University, New York, NY USA

**Keywords:** Deep learning, Segmentation, Infant neuroimaging, Convolutional neural networks, Hippocampus, Amygdala, Behavioral problems

## Abstract

Infant brain magnetic resonance imaging (MRI) is a promising approach for studying early neurodevelopment. However, segmenting small regions such as limbic structures is challenging due to their low inter-regional contrast and high curvature. MRI studies of the adult brain have successfully applied deep learning techniques to segment limbic structures, and similar deep learning models are being leveraged for infant studies. However, these deep learning-based infant MRI segmentation models have generally been derived from small datasets, and may suffer from generalization problems. Moreover, the accuracy of segmentations derived from these deep learning models relative to more standard Expectation–Maximization approaches has not been characterized. To address these challenges, we leveraged a large, public infant MRI dataset (*n* = 473) and the transfer-learning technique to first pre-train a deep convolutional neural network model on two limbic structures: amygdala and hippocampus. Then we used a leave-one-out cross-validation strategy to fine-tune the pre-trained model and evaluated it separately on two independent datasets with manual labels. We term this new approach the **I**nfant **D**eep learning **SEG**mentation Framework (**ID-Seg**). ID-Seg performed well on both datasets with a mean dice similarity score (DSC) of 0.87, a mean intra-class correlation (ICC) of 0.93, and a mean average surface distance (ASD) of 0.31 mm. Compared to the Developmental Human Connectome pipeline (dHCP) pipeline, ID-Seg significantly improved segmentation accuracy. In a third infant MRI dataset (*n* = 50), we used ID-Seg and dHCP separately to estimate amygdala and hippocampus volumes and shapes. The estimates derived from ID-seg, relative to those from the dHCP, showed stronger associations with behavioral problems assessed in these infants at age 2. In sum, ID-Seg consistently performed well on two different datasets with an 0.87 DSC, however, multi-site testing and extension for brain regions beyond the amygdala and hippocampus are still needed.

**Supplementary Information:**

The online version contains supplementary material available at 10.1186/s40708-022-00161-9.

## Introduction

Identifying early neurobiological markers of psychiatric risk is a critical step toward developing targeted early intervention strategies. Brain imaging studies in the early postnatal period are increasingly used to help achieve this goal [1, 2]. Imaging the brain early in life limits postnatal influences, and thus may help isolate, for instance, the impact of prenatal exposures on neurodevelopment. However, an important stumbling block curtailing progress in infant neuroimaging is developing accurate, reliable, and efficient methods for segmenting brain regions on anatomical infant MRI scans.

The amygdala and hippocampus are two candidate brain regions that may help identify psychiatric risk. For example, studies of anxiety and mood disorders, as well as externalizing disorders, have consistently shown that the structure and function of the amygdala and hippocampus, along with other brain regions, are altered. In infants, amygdala–prefrontal functional connectivity is associated with internalizing symptoms at age 2 [3]; prenatal maternal depression is associated with altered white matter connectivity between the amygdala and prefrontal cortex on infant diffusion MRI scans [4]; and maternal anxiety is correlated with slowed development of the hippocampus over the first 6 months of life [5].

Despite the promise of infant neuroimaging, assessing the structure of key brain regions, including the amygdala and hippocampus, remains a challenging task. First, both structures are relatively small in volume, so even minor segmentation errors may lead to significant miscalculations of morphometric estimates. Second, the curvature of both structures, especially of the hippocampus, makes it difficult for automated segmentation software to correctly delineate them from surrounding neural tissue. Third, the amygdala is adjacent to the hippocampus and has low inter-regional contrast. This makes it difficult for one structure to be distinguished from the other. Because of these challenges, segmentation techniques like atlas-based [6, 7], thresholding or clustering [8], are not always accurate.

Publicly available, automatic segmentation pipelines for infant MRI research have been developed and are widely used [9–11]. However, the agreement between these automated techniques and expert manual segmentation has not been extensively tested, and the testing that has been done has been disappointing. For example, one study [12] reported a 0.39 dice similarity score between an automatic method [9] and manual segmentation for the amygdala and hippocampus. Agreement with "ground-truth” manual labels may be worse when automated techniques are applied to datasets acquired from different MRI scanners or imaging protocols. Infant FreeSurfer [11], a novel automated pipeline, can segment the hippocampus and amygdala on T1w scans, but it cannot accommodate T2w MRI images, which are collected by many laboratories doing infant MRI research. While the dHCP pipeline[10] can handle both T1w and T2w, testing of dHCP segmentations against manual segmentations has not been documented.

Deep learning approaches, such as convolutional neural networks (CNN), have been successfully applied in adolescent and adult MRI scans to improve the segmentation accuracy of subcortical regions [13–18]. Variants of CNNs have also been developed for infant research [19–22]. However, the performance of CNNs is directly dependent on the type, quality, and quantity of training data. Limitations in any of these three areas may result in CNN models with poor reproducibility or poor stability across datasets whose acquisition or protocols differ from the training dataset. To address differences across imaging protocols or MRI scanners, transfer-learning has been used to improve CNN-based brain segmentation accuracy for adults [18, 23]. In the deep learning community, transfer-learning refers to a technique to fine-tune a model, which has already been pre-trained on a large dataset, such that the model can accommodate specific domains within smaller datasets. Transfer learning has not been used in infant brain research, but it holds promise for improving the generalization of CNN-based segmentation models.

This study aimed to use several independent infant MRI datasets and a transfer-learning strategy to improve the generalization of CNN-based segmentation models. We termed this new approach the **I**nfant **D**eep learning **SEG**mentation Framework, or "**ID-Seg**”. We quantitatively and rigorously compared amygdala and hippocampus segmentations derived from ID-Seg with “ground-truth” manual segmentations. Specifically, we first pre-trained a classic U-shape convolutional neural network model on a large, public infant MRI dataset (*n* = 473, collected on a Phillips scanner) on two limbic structures: amygdala and hippocampus. Henceforth, we refer to this dataset as the “training dataset”. Then we fine-tuned and evaluated this pre-train CNN model on two independent datasets separately using leave-one-out cross-validation (LOOCV). These two additional datasets both had manual segmentations of the amygdala and hippocampus. One dataset, which included 20 infant MRI scans, was collected on a GE scanner and then segmented by trained analysts in our research group. Henceforth, we refer to this dataset as the “internal dataset”. Another dataset, which included 10 infant MRI scans, was collected on a Siemen scanner and then segmented by an expert from an external research group. Henceforth, we refer to this dataset as the “external dataset”. The segmentation accuracy of ID-Seg against manual segmentations was calculated by three metrics: Dice similarity coefficients (DSC), intra-class correlation (ICC), and average surface distance (ASD). We similarly calculated the segmentation accuracy against manual segmentations of an existing infant segmentation pipeline, the Developmental Human Connectome pipeline (dHCP), which uses an Expectation–Maximization approach. To justify the contribution of the proposed transfer-learning and pre-training strategy, we also conducted an ablation study by comparing the performance from ID-Seg with pre-trained weights to ID-Seg without pre-trained weights.

Lastly, we conducted a proof-of-concept analysis. Detecting brain–behavior relationships is often an important goal in neurodevelopmental research. We therefore tested whether ID-Seg improves the detection of brain–behavior relationships as compared with an existing infant segmentation pipeline, the dHCP. We hypothesized that ID-Seg-derived morphometric measures would provide stronger brain–behavior associations. To test this hypothesis, we included another dataset with 50 T2w infant MRI scans (henceforth termed the “proof-of-concept” dataset) and parent-reported behavioral problems at age 2 as indexed by the Child Behavior Checklist (CBCL) [24]. Brain measures included amygdala and hippocampus volumes and shapes, and behavioral measures included internalizing, externalizing, and total problems.

## Infant MRI datasets

We curated four infant MRI datasets (from 3 different MRI scanners) to test the segmentation performance of ID-Seg. All structural MRI scans went through minimal preprocessing including skull stripping [25] and N4 bias field correction [26]. Demographics and imaging parameters of each dataset are presented in **Table ****1**.

**Table 1:** Demographics and MRI sequence information

	Training DHCP^*^ (*N* = 473)	ECHO-Dataset1^ƒ^ (*N* = 20)	M-CRIB^†^ (*N* = 10)	ECHO-Dataset2^‡^ (*N* = 50)
PMA at scan, weeks	40.65 ± 2.19	46.90 ± 4.14	39.78 ± 1.31	48.06 ± 4.69
Sex				
Female, N(%)	266 (43.8%)	13 (65.0%)	4 (40.0%)	25 (50%)
Male, N (%)	207 (56.2%)	7 (35.0%)	6 (60.0%)	25 (50%)
MRI scanners	3 Philips	3T GE	3T Siemens	3T GE
MRI resolution (mm^3^)	[0.5, 0.5, 0.5]	[0.9, 0.9, 0.9]	[0.63, 0.63, 0.63]	[0.9, 0.9, 0.9]
MRI dimensions	[290,290,203]	[130,256,256]	[304,304,157]	[130, 256, 256]

For quantitative variables, data are presented as mean ± standard deviation unless otherwise noted. PMA: postmenstrual age. ^*^The large training DHCP dataset with corresponding dHCP labels was used to pre-train the model with sufficient data. ^ƒ^The ECHO-Dataset1 dataset was used to test the model’s performance as an internal source. ^†^The M-CRIB dataset was used as an external test dataset to further test the reliability of our proposed deep learning framework. ^‡^The proof-of-concept ECHO-Dataset2 was used to test the association between brain morphometric measures at birth and corresponding CBCL measures at age 2-year-old.

### Training dataset—developmental human connectome (dHCP) project

We included **473** bias-corrected infant T2-weighted (T2w) structural MRI scans from the dHCP v1.0.2 data release The dHCP structural segmentation pipeline [10] generated the bilateral hippocampus and amygdala segmentations.

### Internal dataset—Environmental Influences on Child Health Outcomes (ECHO)-Dataset 1

We used **20** high-quality, term-born infant T2w MRI scans to create our internal dataset. These 20 scans were randomly drawn from the following two Environmental influence on Children’s Health Outcomes (ECHO) studies [27]. Additional information about the two studies, including subject enrollment, imaging parameters, and inclusion/exclusion criteria, can be found in Additional file 1: ECHO Datasets. We manually segmented the bilateral hippocampus and amygdala using a multi-rater method (see Sect. 3.1).

### External dataset—Melbourne Children's Regional Infant Brain (M-CRIB)

For our external dataset, we used a publicly available dataset that includes T2w MRI scans from 10 term-born infants. The manual segmentations were performed by a single rater from the Melbourne group [7]. Additional details about the manual segmentation procedures and imaging parameters can be found elsewhere [7].

### Proof-of-concept dataset—ECHO-Dataset 2

 We examined prospective brain–behavior associations in 50 infants. Participants had T2w MRI scans during infancy and then CBCL assessments completed at age 2 as part of the aforementioned ECHO studies. These infants do not overlap with those of ECHO-Dataset 1. The MRI scans were collected between 1 and 4 months after birth, and then the parent-report CBCL assessments were performed at 2 years of age.

## Materials and methods

**Fig. 1:**
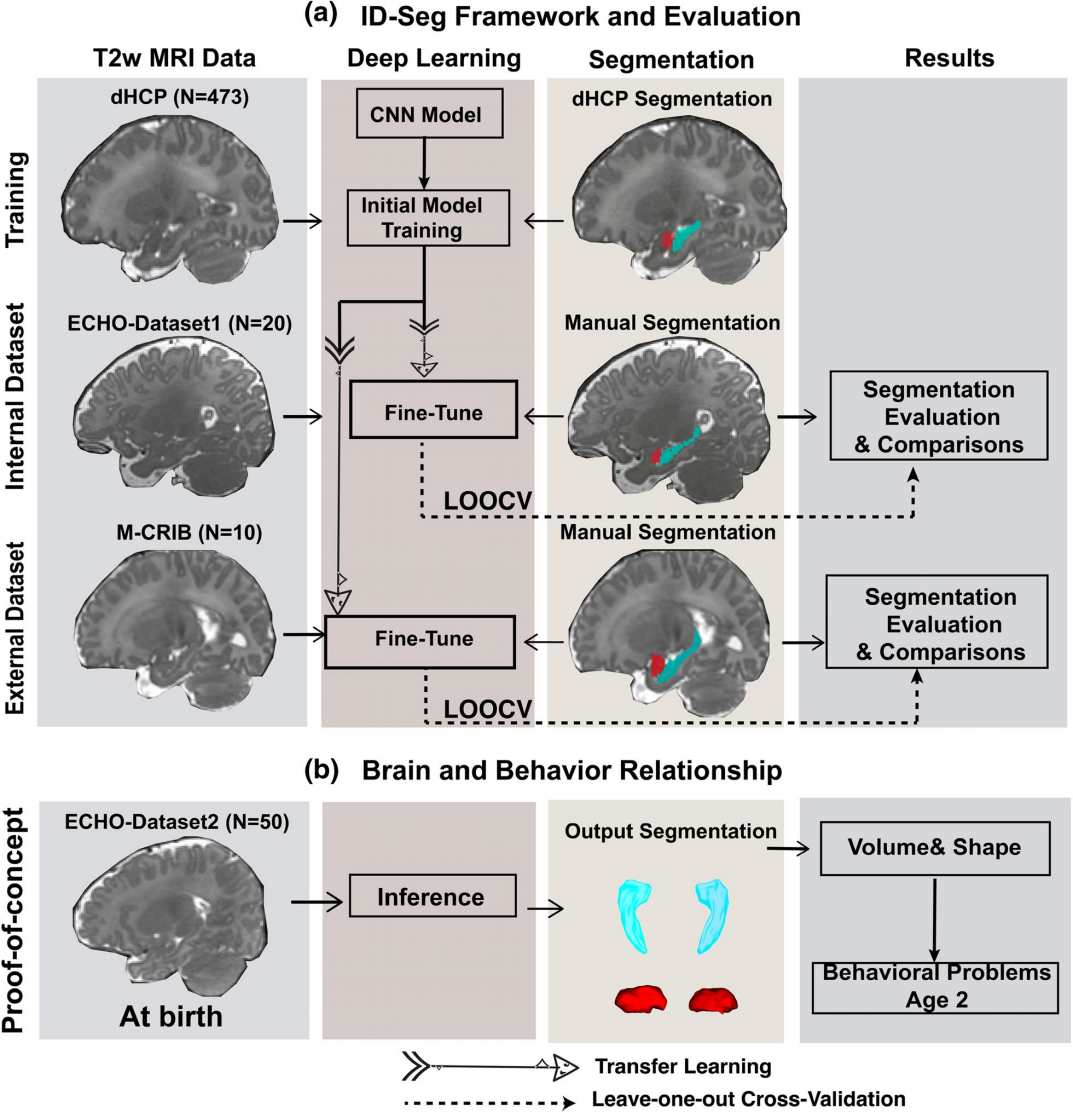
Overview of this study. **a** Using 3 independent infant MRI datasets through a transfer-learning approach, we trained, fine-tuned, and cross-validated a deep-learning segmentation framework (ID-Seg) for hippocampus and amygdala, both with internal and external datasets; **b** we further explored the prospective associations between morphometric measures (left and right hippocampus and amygdala) in infants and behavior problems at age 2. *Cyan color represents segmented hippocampus, and red represents segmented amygdala. *LOOCV* leave-one-out cross-validation

The study contains two modules. First, we built and validated an infant deep learning segmentation framework (**ID-Seg**) to segment the infant hippocampus and amygdala on T2-weighted (T2w) MRI brain scans (Fig. 1a). Second, we conducted a proof-of-concept analysis to explore prospective associations between brain structure in infancy and behavioral problems at age 2 (Fig. 1b).

### Multi-rater manual segmentation

Three research assistants (KS, GKCAA, EB) received instruction and training from a board-certified radiologist (AM) to perform infant hippocampus and amygdala segmentation using ITK-SNAP software [28]. Manual segmentation protocols are available in the Additional file 1: Manual Segmentation Protocols using ITK-SNAP. Inter-rater reliability for these manual tracings was assessed by Dice Similarity Score (DSC) and ensured a minimum 0.6 DSC for each brain region before proceeding. Based on all three raters' manual segmentation, a bilateral reference manual segmentation for the amygdala and hippocampus was generated with the Simultaneous Truth And Performance Level Estimation (STAPLE) algorithm [29]. STAPLE is an expectation–maximization algorithm that estimates the optimal combination of segmentations based on each rater's performance level. We established the "ground truth" segmentation using STAPLE and based on all three study raters instead of only one rater. We visually inspected and edited STAPLE output if needed.

### Infant deep learning segmentation (ID-Seg)

We adopted a transfer-learning approach to train and test ID-Seg on multiple datasets. In Fig. 1a, ID-Seg was initially trained (termed “pre-train” in the AI literature) on our training dataset, consisting of 473 T2w infant MRI scans. Then we tested this trained model on our internal dataset (ECHO-Dataset 1) and external dataset (M-CRIB). For our internal dataset, we used our multi-rater manual segmentation framework as described above to generate manual segmentations; for out external dataset, researchers from an independent group [7] generated manual segmentations and have made these publicly available (https://osf.io/4vthr/). All deep learning models below were written in Python using PyTorch libraries, and relative training procedures were completed in the NVIDIA Geforce Titan RTX GPU workstation. Relevant code is open access via GitHub repository (https://github.com/wangyuncolumbia/ID-Seg-V2).

#### Model architecture

We used a multi-view fully convolutional neural network, the most cited MRI brain segmentation model [18]. This model was initially developed for adult whole-brain segmentation and has been demonstrated to be capable of segmenting small subcortical structures with skip connections and unpooling layers in the decoding path. The flowchart of this model’s architecture can be found in Additional file 1: Fig. S1*,* and more detailed information can be found in the original work [18]. Specifically, we trained three 2D CNN models separately for each of the three principal views (axial, coronal, and sagittal). Of note, each 2D CNN has the same architecture. In the end, we merged predicted probabilities from multi-view models using formula (1) below to calculate the final predicted label for each voxel:

1 $${L}_{Pred }\left(x\right)= argmax \left({\lambda }_{1}{p}_{Axial}\left(x\right)+ {\lambda }_{2} {p}_{Coronal}\left(x\right)+ {\lambda }_{3} {p}_{Sagittal}\left(x\right)\right).$$

In formula (1), ${p}_{Axial}\left(x\right), {p}_{Coronal}{\left(x\right), p}_{Sagittal}(x)$ are the predicted probabilities of a voxel from axial, coronal, and sagittal deep learning models. We set the weights ${\lambda }_{1}, {\lambda }_{2}, {\lambda }_{3}$ to 0.4, 0.4, and 0.2, respectively.

When using this model, there is no fixed dimension requirement for the input size of MRI images, however, the input size should be divisible by 16 because this model consists of 4 down-sampling layers – each layer reduces the image by a factor of 2. Therefore, for each dataset in this project, we changed the size of the input image to meet this divisibility rule by cropping background borders (equally from both sides) or up-sampling the field of view to ensure a fair comparison if the input size were significantly smaller than the training dHCP dataset. Detailed information can be found in Additional file 1:Table S1.

#### Model learning

##### Initial network training

The goal of training ID-Seg on a large training dataset first was to provide robust weight initialization. We randomly split this training dataset into two parts: 80% for training and 20% for validating model performance. As noted above, we applied the dHCP structural segmentation pipeline, which bilaterally segments and labels 87 regions within the infant brain, including the hippocampus and amygdala. We started with these automated segmentations because we reasoned that this large sample would provide strong prior initialization of the network, such that we could then optimally utilize the smaller sample of manually segmented scans to achieve high segmentation accuracy. We anticipated that the segmentations from the automated software (i.e., dHCP) would not be as accurate as the manual annotations. However, these segmentations would allow our model to recognize a wide range of morphological variations in brain structures. This training procedure affords strong prior weights for the ID-Seg network, where robustness to data heterogeneity is enhanced by the diversity of the training dataset (e.g., different scanners and sites). For each 2D model, trainable parameters are 3,520,871 and the total trainable parameters are 10,562,613 for the multi-view 2D model. During the training process, we selected a set of model hyperparameters including epoch, dropout, convolutional kernel size, optimizer, learning rate, loss function, and batch size. We chose an optimal configuration that results in a model that achieves the best performance on the validation dataset. The optimal hyperparameter configuration can be found in Additional file 1: Table S2.

##### Internal fine-tuning and leave-one-out cross-validation

We next applied the initially trained ID-Seg and fine-tuned it on our internal dataset (ECHO-Dataset 1). Specifically, we first passed weights of the initially trained ID-Seg, and we then trained ID-Seg for 5 epochs while only unfreezing the last few layers to prevent propagation errors due to random initialization weights and to save computation time. Lastly, we unfroze all layers and fine-tuned the whole network for another 15 epochs. Similarly, we evaluated ID-Seg's performance against manual segmentations that we conducted on the internal dataset using a multi-rater framework and the leave-one-out cross-validation (LOOCV) technique. The training loss versus epochs plot on this dataset can be found in Additional file 1: Fig. S2a. The hyperparameters used to fine-tune the network, including learning rate, batch size, and epoch number, were 5*10^–4^, 8, and 15, respectively.

##### External fine-tuning and leave-one-out cross-validation

To test the reliability of ID-Seg, we applied ID-Seg to our external dataset (M-CRIB). We evaluated the accuracy of ID-Seg’s segmentations against manual segmentations performed by an independent group. The training loss versus epochs plot on this dataset can be found in Additional file 1: Fig. S2b. We used similar hyperparameters as in our internal fine-tuning.

For comparison, we also segmented the internal and external datasets with (1) ID-Seg without pre-training on dHCP dataset; and (2) an automated pipeline, the dHCP, that uses an Expectation–Maximization approach, rather than deep learning.

### Segmentation evaluations and comparisons

We calculated three commonly used evaluation metrics to compare the segmentation output of our ID-Seg against manual segmentations: Dice similarity coefficient (DSC), intra-class correlation (ICC), and average surface distance (ASD). DSC is a metric used to calculate the similarity between two images and measure the overlap across the two images [30]. ICC is a measure of consistency between two raters, or in this case, two segmented images [31]. ASD is a surface-based metric and measures the average Hausdorff Distance over all points between surfaces of a prediction structure (i.e., ID-Seg’s segmentation of the hippocampus and amygdala) and the “‘ground truth”’ (i.e., manually segmented hippocampus and amygdala). Relevant code can be found at https://github.com/deepmind/surface-distance. For each structure, we calculated DSC, ICC, and ASD to compare the output of each method with manual segmentation. One-way ANOVA tests were used to compare the accuracy of three methods: ID-Seg without pre-training, ID-Seg with pre-training and the dHCP pipeline based on DSC, ICC, and ASD.

### Brain and behavior relationship—a proof-of-concept analysis

#### Volumetric and shape analysis for the hippocampus and amygdala

We applied the optimized version of ID-Seg to directly segment infant MRI scans in our proof-of-concept dataset (ECHO-Dataset 2)—that is, infant MRI scans that were not used in any of the previous training/testing procedures (Fig. 1b). Using ID-Seg, we calculated volumetric and shape measurements for the bilateral hippocampus and amygdala. The volume (in mm^3^) of each region was adjusted with respect to total brain volume. Then, we performed shape analysis for each structure using SlicerSALT software (Kitware, Inc., United States). Here, we used an average spherical harmonics description (SPHARM) to represent the shape measurements of a 3D structure [32].

#### Brain and behavior relationship

We conducted Spearman rank partial correlation analysis to examine prospective associations between morphometric measures of the hippocampus and amygdala at infancy with behavioral outcomes at age 2. The behavior outcomes included internalizing, externalizing, and total behavioral problems and were assessed using the T score from parent-report CBCL. We adjusted for postmenstrual age of the infant at MRI scan, maternal education, and maternal post-partum mood symptoms (as indexed by the 10-item Edinburgh Postnatal Depression Scale [33]). Infant sex was not adjusted for because this is already accounted for in CBCL T scores.

## Results

### Multi-rater manual segmentation

With our internal dataset ECHO-Dataset1 (*n* = 20*)*, our multi-rater manual segmentation framework (see Sect. 3.1) generated “ground-truth” segmentation of the bilateral hippocampus and amygdala. For each structure, the mean and standard deviation DSC was 0.78 (0.05) for right hippocampus, 0.77 (0.05) for left hippocampus, 0.73 (0.05) for right amygdala, and 0.74 (0.06) for left amygdala. The inter-rater agreement across all structures was an average DSC of 0.76**.**

Detailed inter-rater agreement is summarized in Additional file 1: Table S1**.**

### Segmentation evaluations and comparisons

**Fig. 2:**
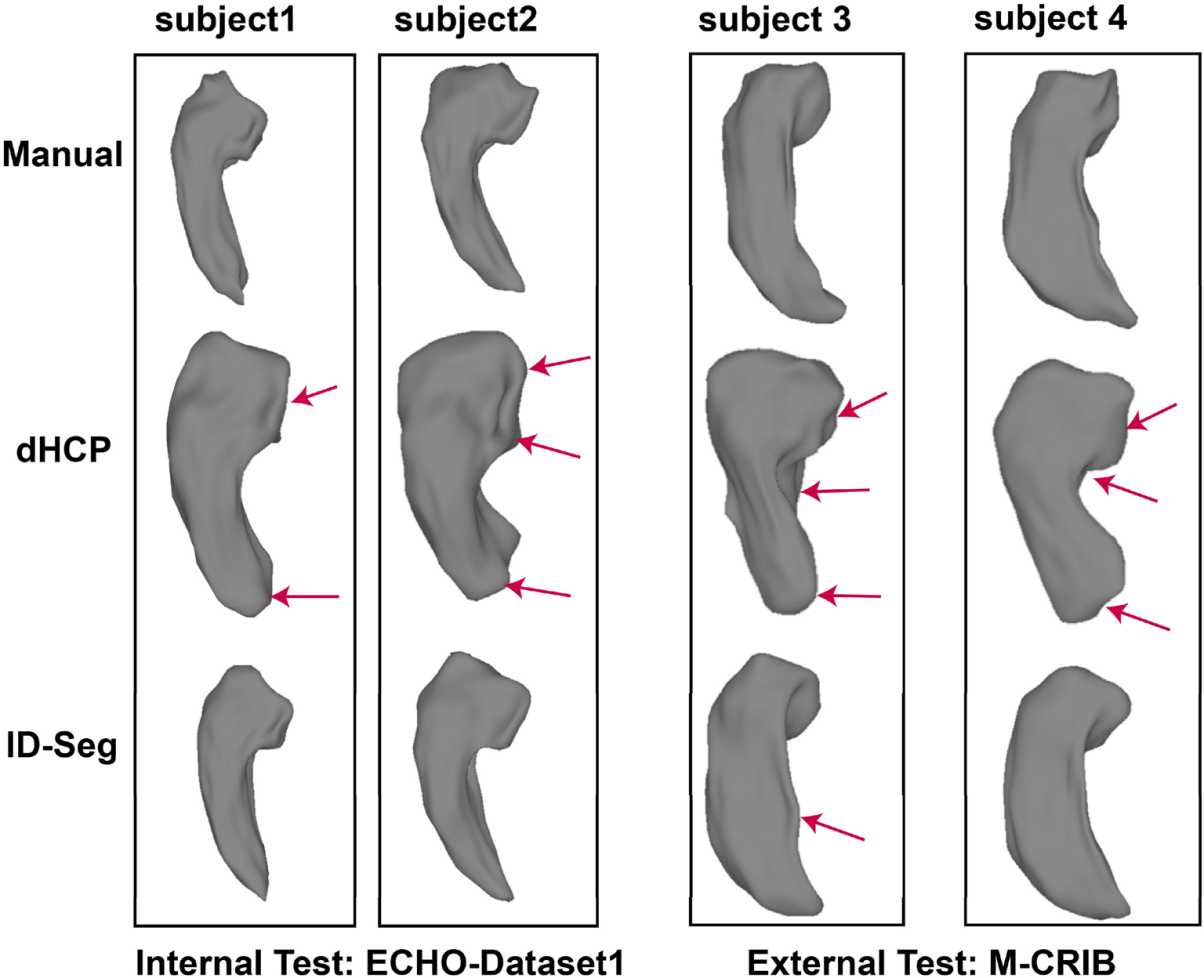
Visual comparisons between the “ground-truth” manual, dHCP, and ID-Seg segmentations for the left hippocampus’s 3D shape using our internal (ECHO-Dataset1) and external (M-CRIB) datasets, respectively. Red arrow highlights the areas with notable differences

On our internal (ECHO-Dataset1) and external (M-CRIB) datasets, the accuracy of ID-Seg was better than that of the dHCP pipeline. Specifically ID-Seg generated segmentations were more accurate relative “ground truth” manual segmentations than those generated by the dHCP pipeline. This was true across all three metrics of accuracy: DCS, ICC, and ASD. Complete statistical results are available in Table 2. As shown in Fig. 2, as compared with the dHCP pipeline, segmentation from ID-Seg generally had smaller volumes, smoother surface, and shapes that more closely match expected anatomical features.

**Table 2:** Segmentation evaluations and comparisons

Dataset	Metric	Region	Segmentation method			F	p.adj
			dHCP	ID-Seg without pre-training	ID-Seg with pre-training		
Internal dataset: ECHO-Dataset1	DSC	L Amyg	0.79 (0.12)	0.76(0.07)	0.86(0.03)	8.81	0.001
(n = 20)		L Hippo	0.76 (0.09)	0.75 (0.08)	0.87 (0.03)	14.10	< 10^−4^
		R Amy	0.77 (0.14)	0.76 (0.08)	0.86 (0.03)	6.31	0.003
		R Hippo	0.74 (0.15)	0.76 (0.08)	0.87 (0.03)	10.67	< 10^−4^
	ICC	L Amyg	0.87 (0.09)	0.86 (0.05)	0.92 (0.02)	3.41	0.05
		L Hippo	0.86 (0.08)	0.85 (0.06)	0.93 (0.02)	4.75	0.024
		R Amyg	0.86 (0.13)	0.86 (0.05)	0.92 (0.02)	3.79	0.05
		R Hippo	0.84 (0.13)	0.86 (0.05)	0.93 (0.01)	5.89	0.02
	ASD	L Amyg	0.49 (0.34)	0.41 (0.09)	0.32 (0.11)	5.89	0.007
		L Hippo	0.60 (0.37)	0.61 (0.59)	0.26 (0.11)	10.38	0.001
		R Amyg	0.53 (0.47)	0.79 (1.3)	0.31 (0.09)	3.97	0.024
		R Hippo	0.65 (0.43)	0.66 (0.57)	0.26 (0.11)	6.43	0.006
External dataset: M-CRIB	DSC	L Amyg	0.73 (0.02)	0.81 (0.03)	0.88 (0.02)	184.47	< 10^−4^
(n = 10)		L Hippo	0.67 (0.04)	0.81 (0.03)	0.88 (0.03)	156.2	< 10^−4^
		R Amyg	0.67 (0.03)	0.83 (0.04)	0.87 (0.02)	68.78	< 10^−4^
		R Hippo	0.60 (0.04)	0.83 (0.03)	0.87 (0.03)	109.61	< 10^−4^
	ICC	L Amyg	0.86 (0.05)	0.90 (0.01)	0.93(0.01)	195.48	< 10^−4^
		L Hippo	0.83 (0.06)	0.91 (0.02)	0.94(0.01)	393.73	< 10^−4^
		R Amyg	0.83 (0.06)	0.90 (0.02)	0.93(0.01)	88.13	< 10^−4^
		R Hippo	0.78 (0.09)	0.91 (0.01)	0.93(0.02)	373.74	< 10^−4^
	ASD	L Amyg	0.94 (0.13)	0.55 (0.06)	0.36 (0.11)	190.95	< 10^−4^
		L Hippo	2.5 (0.29)	0.37 (0.17)	0.25 (0.06)	158.59	< 10^−4^
		R Amyg	1.1 (0.11)	0.45 (0.09)	0.41 (0.09)	70.13	< 10^–4^
		R Hippo	3.3 (0.44)	0.41 (0.18)	0.28 (0.06)	112.71	< 10^–4^

*DSC* Dice similarity coefficients, *ICC* intra-class correlation, *ASD* average surface distance, measured in mm. Segmentation metrics for each method are shown in mean (standard deviation). Higher DSC and ICC, and lower ASD indicate better segmentation accuracy. *Amyg* Amygdala, *Hippo* hippocampus; *L* left; *R* right

With ID-Seg with pre-training, on our internal dataset ECHO-Dataset1, the average and standard deviation DSC of ID-Seg across four structures was 0.86 (0.03); ICC was 0.93 (0.02);ASD was 0.29 (0.11) mm. On the external dataset M-CRIB, ID-Seg’s performance was similar: 0.87 (0.02) for DSC, 0.93 (0.01) for ICC, and 0.32 (0.10) for ASD. With the dHCP segmentation pipeline, on our internal dataset ECHO-Dataset1, dHCP’s average DSC was 0.77 (0.13), average ICC was 0.86 (0.11) and dHCP’s average ASD was 0.57 (0.40). On the external dataset M-CRIB, we noticed the performance of dHCP significantly dropped: 0.66 (0.06) for DSC, 0.79 (0.04) for ICC, and 2.0 (1.1) mm for ASD. Each feature’s mean and standard deviation can be found in Table 2**.**

We also compared ID-Seg’s results with and without pre-trained weights. As expected, the results of ID-Seg without pre-trained weights were less accurate as compared to ID-Seg with pre-trained weights.

### Brain at birth and behavioral problems at age 2

**Fig. 3:**
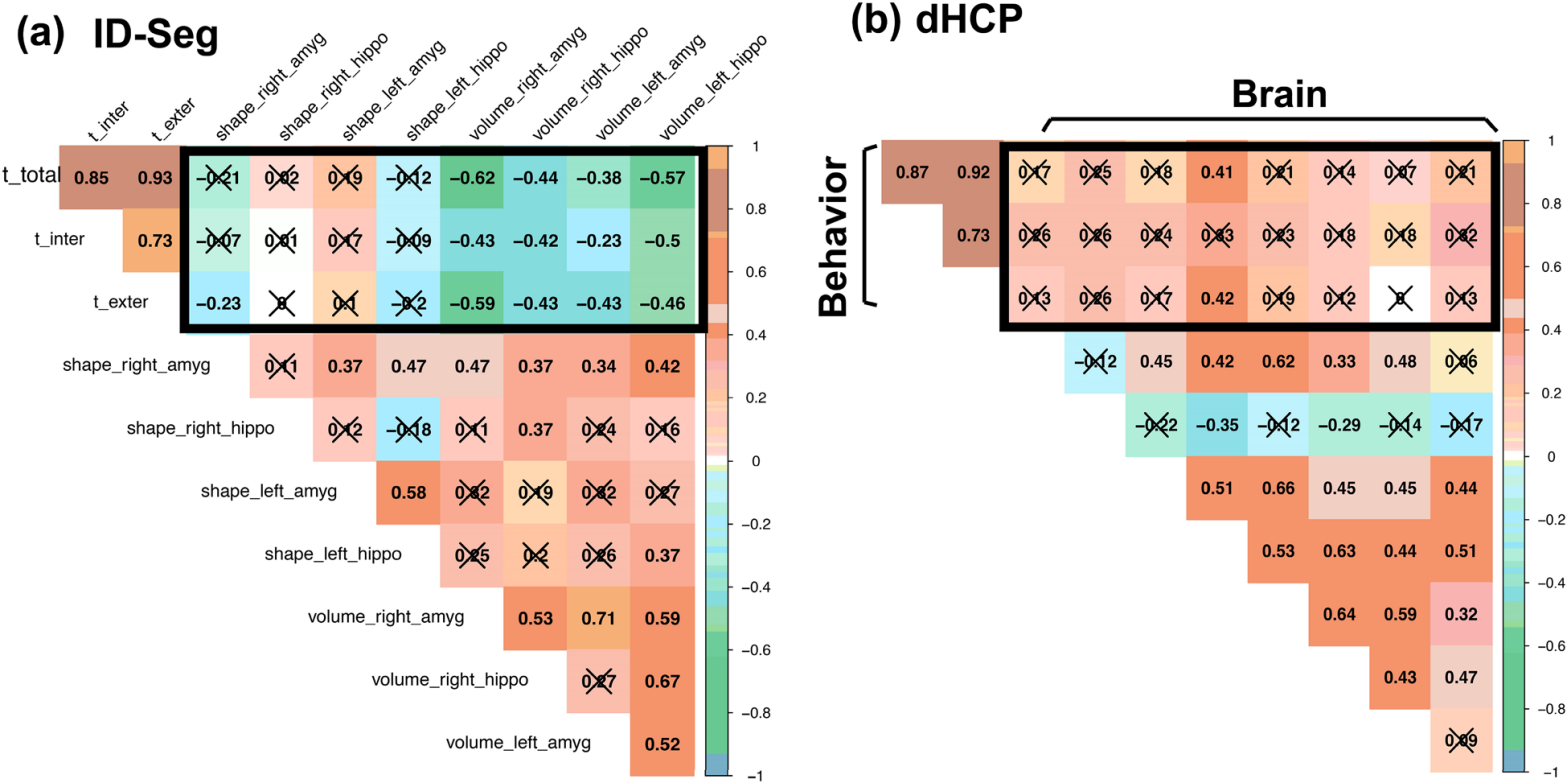
Brain–behavior relationships (in black rectangles) for **a** ID-Seg, **b** dHCP. **X** indicates that the p value of spearman correlation is not significant at the threshold of *p* = 0.05. *t_total* CBCL total problems T score, *t_inter* CBCL internalizing problems T score, *t_exter* CBCL externalizing problems T score, *amyg* amygdala, *hippo* hippocampus

In the proof-of-concept dataset (ECHO-Dataset2), mean and standard deviation T scores on the CBCL at child age 2 were 48.3 (10.6) for total problems, 47.6 (9.12) for internalizing problems, and 48.2 (10.9) for externalizing problems. As seen from Fig. 3a, we identified multiple significant correlations (13 out of 24) between ID-Seg derived brain features and age 2 behavioral outcomes. For example, we found significant correlations between the volume of right amygdala and CBCL total problems, rho(47) = -0.62, *p* < 10^–3^; internalizing problems, rho(47) = − 0.43, *p* < 10^–3^; and externalizing problems, rho(47) = -0.59, *p* < 10^–3^. Conversely, we detected on two significant correlations between dHCP-derived brain features and age 2 behavioral outcomes (Fig. 3b).

## Discussion

In this study, we pre-trained a 3D deep learning infant segmentation (ID-Seg) model on a large sample of infant MRI scans. Using a transfer-learning technique, we then fine-tuned and evaluated the performance of ID-Seg on two datasets with manual segmentations. We found that ID-Seg had a high degree of segmentation accuracy. Lastly, in a proof-of-concept analysis, ID-Seg was more effective in detecting brain–behavior associations than an existing infant MRI pipeline.

Human neuroimaging research requires reliable, efficient and accurate methods for segmenting structural brain MRI scans. This is particularly important for large, multi-site studies that obtain data from several different MRI scanners and use large sample sizes. For example, studies such as the Adolescent Brain Cognitive Development (ABCD) includes over 10,000 MRI scans, collected across multiple sites. Progress toward this goal of optimizing structural MRI segmentation has been achieved for studies of adults and youth. However, structural MRI scans of infants requires special consideration because of the marked differences in tissue contrast in the infant relative to adult or child brain. For example, imaging pipelines such as FreeSurfer that are widely used in adult and youth studies are not able to segment common subcortical structures on infant structural MRI scans. The NIH has recently expanded its support of infant MRI research with the Healthy Brain and Child Development Study (HBCD) [34], that launched in 2021 and aims to obtain more than 10,000 infant MRI scans. The need for reliable, efficient and accurate methods for segmenting infant brain MRI data will continue to grow.

Currently, only two publicly available automated pipelines are capable of infant subcortical segmentation—infant FreeSurfer [11] and the dHCP pipeline. Both offer significant advances to the field, yet they also have limitations. The Infant FreeSurfer pipeline can be used only for T1w scans, yet many structural MRI studies in infant research prefer T2w images because they offer clearer grey–white boundaries. The dHCP pipeline can segment T2w images; however, validation of dHCP segmentation against the “gold-standard” manual segmentations has been limited. It is worth to mention that the manual segmentation protocols between dHCP and Infant Freesurfer are significantly different. The dHCP also requires a high level of computing power and can take several hours to segment a single infant MRI scans, limiting it practical utility for large scale scales such as the aforementioned HBCD.

As an additional option for infant subcortical segmentation, we offer ID-Seg, which combines deep learning and a transfer-learning method, as a high-efficiency, precise, and reliable way to segment the amygdala and hippocampus in the infant brain. Because infant Freesurfer requires T1w scans, we did not compare ID-Seg with infant Freesurfer, We did, however, compare ID-Seg with the dHCP pipeline. We found that structures segmented by ID-Seg had a high degree of segmentation accuracy. Specifically, ID-Seg-derived segmentations of the amygdala and hippocampus were similar to the “ground truth” segmentations done by expert manual raters, and based on three different metrics of accuracy seemed to generate more accurate segmentations than the dHCP pipeline.

There are several possible reasons why the segmentation accuracy of dHCP pipeline is lower than ID-Seg when segmenting limbic structures. First, the dHCP segmentation pipeline was developed based on 20 manually segmented infant brain scans. However, 15 of these were preterm infants. Our work used samples from full-term infants. Numerous studies have previously shown significant structural brain differences in pre- and full-term infants [35, 36]. Second, MRI scans used in our work were obtained from multiple MRI vendors, including GE, Siemens, and Philips. This allowed ID-Seg to learn and adapt to the idiosyncratic features of specific MRI vendors. The dHCP pipeline may have suffered from lower segmentation accuracy due to scanner differences. Third, raters from both internal and external dataset followed the Desikan–Killiany–Tourville protocol [37] to manually segment subcortical regions and our ID-Seg was capable of learning this protocol during the fine-tuning process. However, the protocol for dHCP pipeline[38] is different, potentially leading to lower segmentation accuracy.

Moderate negative associations were observed between ID-Seg-derived limbic structures and parent-reported behavioral problems at age 2. These inverse associations indicated that a smaller hippocampus and amygdala in infancy correlated with more behavioral problems at age 2. The results from this proof-of-concept analysis were from a small sample (*n* = 50), and thus require replication. However, the findings suggest that quantifying morphometrics from limbic substrates may offer valuable insights into future psychiatric impairment. Moreover, these brain–behavior associations were detected more often with ID-Seg-derived limbic segmentations relative to dHCP-derived limbic segmentations. This is consistent with our finding that ID-Seg segmentations were more accurate than dHCP-derived segmentations when compared with “ground truth” manual segmentations. That is, we suspect that brain–behavior association were more often detected with ID-Seg because of its ability to generate accurate segmentations.

Although the reliability and accuracy of ID-Seg are promising, it is important to be aware of limitations. First, ID-Seg can only segment the amygdala and hippocampus and it cannot segment hippocampus and amygdala subfields. Second, ID-Seg has not been tested in infants of different ages (e.g., 4–6 months). Third, ID-Seg has not been tested on T1w MRI scans, nor has it been compared with infant Freesurfer. An indirect future comparison is possible with novel multi-model infant MRI synthesizing models [39]. For example, a T1w scan could be synthesized from T2w and then fed into infant Freesurfer pipeline. Forth, the ground-truth manually segmented dataset, against which we determined the accuracy of ID-Seg, was small. Novel methods, such as SparseGT [40], can be potentially used to save manual workload and expense for generating ground-truth labels. Fifth, ID-Seg framework only adopted an existing popular deep learning architecture. Future work will need to continue developing ID-Seg (e.g., adding domain adaptation methods [41, 42] and T1-to-T2w MRI synthesizing [39]) and expand the ground-truth manual datasets to include infants of different ages, subregions of amygdala and hippocampus, and other subcortical regions (e.g., caudate, putamen). Lastly, in our proof-of-concept analysis, we found that morphometric measures derived from ID-Seg provided moderate brain–behavior associations; however, this was based on a small sample. These brain–behavior findings need to be replicated in larger, independent samples.

In sum, using transfer-learning we adopted a classic deep learning architecture for infant MRI segmentation of the hippocampus and amygdala. Our findings suggest that deep learning architectures may be used to improve the segmentation accuracy of infant MRI scans. Subsequent research can build upon this and continue to apply deep learning to meet the growing need for reliable, accurate, and fast segmentation of infant MRI scans.

## Supplementary Information

**Additional file 1.** Additional materials, tables and figures.

## Data Availability

The training dHCP dataset used during this work is open access and is available upon request from dHCP Consortium. M-CRIB is publicly available on Open Science Framework, https://osf.io/4vthr/. ECHO-Dataset1 will be accessible upon request.

## Code availability

The code used in this paper is publicly available at GitHub, https://github.com/wangyuncolumbia/ID-Seg-V2.
